# Effects of *Lactobacillus reuteri* DSM 17938 in preterm infants: a double-blinded randomized controlled study

**DOI:** 10.1186/s13052-019-0716-9

**Published:** 2019-11-09

**Authors:** Xuewei Cui, Yongyan Shi, Siyang Gao, Xindong Xue, Jianhua Fu

**Affiliations:** Department of Neonatology, Shengjing Hospital, China Medical University, Shenyang, China

**Keywords:** Preterm infant, *Lactobacillus reuteri*, Feeding tolerance, Growth, Infection prevention

## Abstract

**Background:**

Preterm infants have immature gastrointestinal tracts and poor immunity. In this study, the effects of *Lactobacillus reuteri* DSM 17938 first on early feeding tolerance, growth, and second on infection prevention in preterm infants were evaluated.

**Methods:**

One hundred fourteen formula-fed preterm infants with a gestational age between 30 weeks and 37 weeks, and a birth weight between 1500 and 2000 g were enrolled; 57 in the intervention and 57 in the control group:the intervention group was given a dose of 1 × 10^8^ colony-forming units (5 drops) of *L. reuteri* DSM 17938 once daily, beginning with the first feeding until discharge. The control group did not receive probiotics. Early feeding tolerance (as time to full enterla feeding and number of reflux), growth, incidences of sepsis, localized infection, NEC, and adverse effects were recorded for both groups.

**Results:**

The number of Daily reflux episodes (times/d) was lower (2.18 ± 0.83 vs. 3.77 ± 0.66, *P* < 0.01) and time to full enteral feedings (120 mL/kg/d) (9.95 ± 2.46 d vs. 13.80 ± 3.47 d, *P* < 0.05) was shorter in the intervention group. Average daily weight gain (14.55 ± 3.07 g/d vs. 10.12 ± 2.80 g/d), head circumference increas e(0.0760 ± 0.0157 cm/d vs. 0.0681 ± 0.0108 cm/d), and body length increase (0.1878 ± 0.0151 cm/d vs. 0.1756 ± 0.0166 cm/d) of the intervention group were higher (*P* < 0.01). There were no significant differences in the incidences of sepsis (4.44% vs. 8.33%), localized infection (6.67% vs. 8.33%), or NEC (2.22% vs. 10.42%) between the 2 groups (*P* > 0.05). The number of daily defecations (times/d) in the intervention group was higher (3.08 ± 0.33 vs. 2.29 ± 0.20, *P* < 0.01) and the length of hospital stay was shorter than that in the control group (20.60 ± 5.36 d vs. 23.75 ± 8.57 d, *P* < 0.05). No adverse effects were noted among the infants receiving *L. reuteri*.

**Conclusion:**

*L. reuteri* may be an useful tool in improving early feeding tolerance in preterm infants, promoting growth, increasing the frequency of defecation, and shortening the length of hospital stay.

**Trial registration:**

ChiCTR, ChiCTR1900025590. Registered 1 February 2019- Retrospectively registered, http://www.chictr.org.cn/listbycreater.aspx.

## Background

The survival rate of preterm infants has increased substantially in recent years. Preterm infants, due to their immature development, are characterized by underdeveloped gastrointestinal tracts, delayed colonization of intestinal flora, and low immune function. Therefore, preterm infants are prone to feeding intolerance, infection, and even neonatal necrotizing enterocolitis (NEC), all of which affect growth and quality of life [1].

Probiotics are living microorganisms, which, when administered in adequate amounts, can confer a health benefit to the host [2]. As a microecological preparation, probiotics seem to be useful in nourishing the intestines, adjusting the microbiota, ameliorating immunity and reducing inflammation. In 2017, the World Gastroenterology Organization updated global guidelines about the use of probiotics and prebiotics. A large amount of clinical trials confirmed that probiotics have a beneficial effect on the prevention and treatment of digestive diseases [3]. The clinical application of probiotics in neonates is increasingly widespread for many indications, including the prevention and treatment of feeding intolerance, diarrhea, NEC, neonatal jaundice, and allergic diseases [4].

*Lactobacillus reuteri* (*L. reuteri*) DSM 17938 is a progeny strain of *L. reuteri* ATCC 55730, which was originally extracted from the breast milk of Peruvian women living in the Andes. In 2007, 2 plasmids with antibiotic resistance (tetracycline and lincomycin) were removed in order to enhance safety, and the modified strain was stored at The German Center for the Conservation of Microbial Species, also called the DSMZ, and named *L. reuteri* DSM 17938. This strain can live throughout the gastrointestinal tract and colonize in normal human gastric bodies, including the gastric antrum, duodenum, and ileum [5]. In one study, its colonization required continuous supplementation for 7 days, and the highest colonization rate was reached at 21 days; after discontinuing supplementation, the colonization rate decreased significantly at 1 week and was undetectable at 2 months [6]. The role of *L. reuteri* in the effective treatment of infantile colic is clearly illustrated [7, 8], and it has been widely used in the prevention and treatment of infantile reflux, functional constipation, and acute gastroenteritis. However, research of the effects of *L. reuteri* on the clinical course in preterm infants is limited.

The aim of the study was to investigate the efficacy of *L. reuteri* in early feeding tolerance, growth, infection prevention, and other aspects of preterm infant development.

## Materials and methods

### Patients

Preterm infants admitted to the First Neonatal Ward of Shengjing Hospital of China Medical University from January 2017 to June 2018 were enrolled for this double-blinded randomized controlled study. Inclusion criteria: formula-fed preterm infants admitted within 12 h of birth whose gestational age ≥ 30 and < 37 weeks; birthweight≥1500 g and ≤ 2000 g with vital sign and hemodynamic parameters stable. Exclusion criteria: congenital diseases, expected hospitalization less than 2 weeks and maternal or neonatal antibiotics or other probiotics before admission.

The study was approved by the Medical Ethics Committee of Shengjing Hospital of China Medical University. Informed consent was obtained from the infants’ parents. Randomization was conducted according to a random computer-determined allocation order considering gestational age.

### Study design

After admission, routine treatment and nursing support were provided for all infants enrolled according to their conditions. Formula milk feeding (provided by the hospital, 335 kJ/100 mL, Abbott Laboratories, USA) was given to the preterm infants after stabilization, started with 20 mL/kg/d and was defined as full enteral feeding when 120 mL/kg/d were reached. Increment were 10–20 mL/kg/d depending on the gestational age of the infant. The amount of feeding was advanced if tolerated with 10–20 mL/kg/d. This feeding policy was not altered during the study period. Parenteral nutrition was supplemented for undernutrition, as defined by the “Guidelines for Clinical Application of Neonatal Nutrition Support in China” [9]. *L. reuteri* DSM 17938 (Biogaia AB, Stockholm, Sweden) was administered to the preterm infants in the intervention group at a dose of 1 × 10^8^ colony-forming units (5 drops) once daily, beginning with the first feeding until discharge from the hospital. For infants who were fed orally, 5 drops were instilled to the posterior oropharynx of the infants after suctioning oral secretions. For infants without oral feeds, 5 drops were administered through a gastric tube followed by a flush of 0.5 mL of sterile water. The minimum duration of the intervention was 7 days. No probiotics were administered to the control group. If enteral feeding was stopped due to feeding intolerance such as increased abdominal girth, emesis, gastric residual of 25% of the previous feed volume or NEC during hospitalization, *L. reuteri* was stopped and resumed after feeding resumed. Blinding was possible because the nurses who administered *L. reuteri* to the infants were not involved in the daily care and the attending neonatal team was unaware of the randomization assignments.

### Primary and secondary outcomes

Primary outcomes of this study were feeding tolerance and growth in preterm infants. Secondary outcome was infection prevention. To assess feeding tolerance, the time to full enteral feedings (TFF) and number of reflux episodes were recorded. To assess growth indicators, we measured the growth of body weight, body length, and head circumference, calculating the difference of these parameters at the end toward the beginning of study period, then we divided them as daily increase. To assess infection prevention, incidences of nosocomial infection and NEC were recorded. Adverse effects, including culture-proven sepsis, flatulence and diarrhoea were also recorded.

### Definitions and diagnostic criteria

Reflux was defined as the passage of refluxed gastric contents into the oral pharynx [10]. Nosocomial infection was defined as an infection occurring after 48 h of hospitalization, including infections that occurred during hospitalization and infections that occurred after discharge from pathogens to which infants were exposed in the hospital [11]. NEC was defined as acute necrotizing intestinal disease with abdominal distension, vomiting, and hematochezia as the main symptoms. NEC may be caused by various factors in the perinatal period; it should be diagnosed according to clinical manifestations and abdominal X-rays and should be categorized by modified Bell’s classification [12].

### Statistical analysis

SPSS Statistics version 24 statistical software was used for statistical analysis. The measurement data are presented as means ± standard deviations. The t-test was used for comparisons between groups. Categorical data were compared by the χ^2^ test. A *p*-value of less than 0.05 was considered statistically significant. This study was registered at the website http://www.chictr.org.cn/listbycreater.aspx under the number ChiCTR1900025590.

## Results

### Patient description

**Fig. 1:**
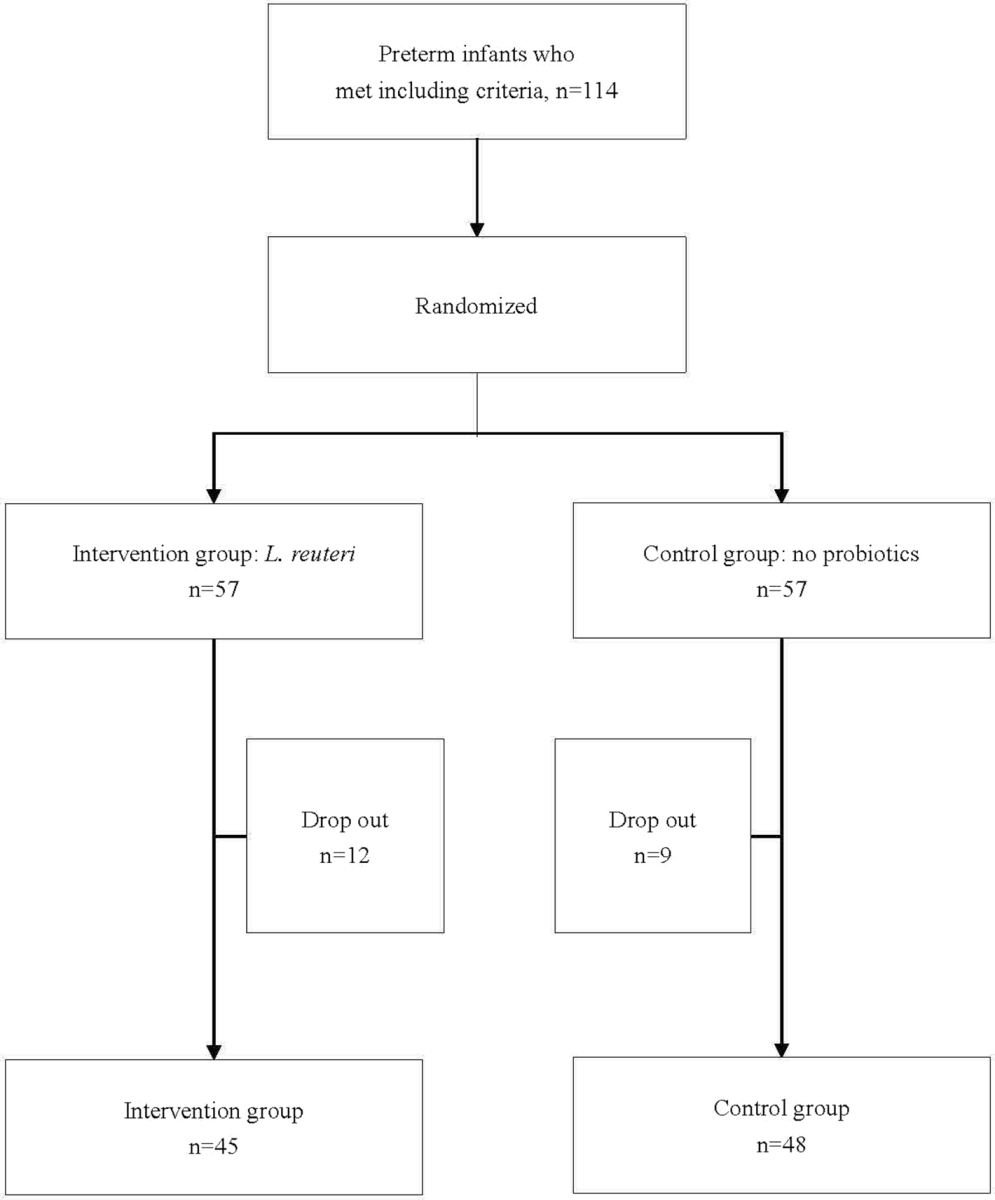
Study flow chart

A total of 114 cases were eventually enrolled and randomly allocated in the study. 57 patients received *L. reuteri* and 57 neonates were included in the control group. In all, 21 (18%) patients were excluded because of major congenital malformations and the using of other probiotics (12 [21.1%] in the intervention group and 9 [15.8%] in the control group). Finally, 45 cases could be analyzed in the intervention group and 48 cases in the control group (Fig. 1).

The characteristics of the preterm infants at study inclusion are shown in Table 1. There were no significant differences in gender, gestational age, birth weight, or Apgar score between the 2 groups.

**Table 1:** Comparison of the Characteristics Between the 2 Groups of Preterm Infants

Group	Intervention group	Control group	*P*
*n*	45	48	
Gender (M/F)	25/20	20/28	
Gestational age ($\overline{x}$_(sd), weeks_)	32.85 (1.39)	32.56 (1.41)	0.3206
5 min Apgar score ($\overline{x}$_(sd)_)	9.29 (0.84)	9.29 (0.82)	0.9872
Birth weight ($\overline{x}$_(sd),_ g)	1682 (109.03)	1714 (127.11)	0.1984

$\overline{x}$(sd)*,* means (standard deviation)

### Primary and secondary outcomes

The study outcomes observed in the two study groups are shown and compared in Table 2.

**Table 2:** Comparison of Feeding Tolerance, Growth, and Infection incidence Between the 2 Groups

	Intervention group	Control group	*t*	*X*^*2*^	*P*
Feeding tolerance					
Reflux (times/d)	2.18 ± 0.83	3.77 ± 0.66	−10.250		0.000
TFF (d)	9.95 ± 2.46	13.80 ± 3.47	−2.874		0.015
Growth					
Weight gain (g/d)	14.55 ± 3.07	10.12 ± 2.80	7.269		0.000
HC increase (cm/d)	0.0760 ± 0.0157	0.0681 ± 0.0108	2.794		0.007
Body length increase (cm/d)	0.1878 ± 0.0151	0.1756 ± 0.0166	3.687		0.000
Infection incidence					
Sepsis	4.44%	8.33%		0.582	0.446
Localized infection	6.67%	8.33%		0.093	0.761
NEC	2.22%	10.42%		2.584	0.108
Other indicators					
Defecation (times/d)	3.08 ± 0.33	2.29 ± 0.20	13.891		0.006
Hospital stay (d)	20.60 ± 5.36	23.75 ± 8.57	−2.139		0.036

*HC* head circumference, *NEC* neonatal necrotizing enterocolitis, *TFF* time to full enteral feedings

#### Feeding tolerance

The number of daily reflux episodes (times/d) in the intervention group was significantly lower than that in the control group (2.18 ± 0.83 vs. 3.77 ± 0.66, *P* < 0.01). TFF in the intervention group was significantly shorter than that in the control group (9.95 ± 2.46 d vs.13.80 ± 3.47 d, *P* < 0.05).

#### Growth

The average daily weight gain (14.55 ± 3.07 g/d vs. 10.12 ± 2.80 g/d), daily head circumference growth (0.0760 ± 0.0157 cm/d vs. 0.0681 ± 0.0108 cm/d), and body length increase (0.1878 ± 0.0151 cm/d vs. 0.1756 ± 0.0166 cm/d) of the preterm infants in the intervention group were significantly higher than those of the control group (*P* < 0.01).

#### Infection

Nosocomial infection could be divided into sepsis and localized infection. In this study, there were 2 cases of sepsis and 3 cases of localized infection (2 cases of neonatal pneumonia, 1 case of urinary tract infection) in the intervention group while 4 cases of sepsis and 4 cases of localized infection (3 cases of neonatal pneumonia, 1 case of urinary tract infection) in the control group. There was no significant difference in the incidences of sepsis (4.44% vs. 8.33%), localized infection (6.67% vs. 8.33%) or NEC (2.22% vs. 10.42%) between the 2 groups (*P* > 0.05).

#### Other indicators

The number of daily defecations in the intervention group was 3.08 ± 0.33, which was 2.29 ± 0.20 in the control group. The length of hospital stay in the intervention group was shorter than that in the control group (20.60 ± 5.36 d vs. 23.75 ± 8.57 d, *P* < 0.05). The preterm infants in the intervention group had no adverse effects while receiving *L. reuteri*.

## Discussion

Preterm infants have poor sucking and swallowing abilities, immature digestive systems, insufficient gastrointestinal motility, and low gastrointestinal mucosal barrier function, and they are prone to feeding intolerance such as regurgitation, vomiting, abdominal distension, and gastric retention during feeding [13]. Studies have shown that probiotics could stimulate the secretion of gastrin and motilin, promote gastrointestinal motility, reduce the incidence of feeding intolerance, and shorten the time to achieve total enteral nutrition [14]. Reflux refers to the reverse movement of gastric contents, usually referred to gastroesophageal reflux (GOR). GOR is a prominent condition among preterm infants. Symptoms such as apnoea, bradycardia, vomiting, poor weight gain and irritability have been attributable to GOR, which is called gastroesophageal reflux disease (GORD), when symptoms are severe [15]. Experimental data showed that *L. reuteri* can promote gastric motility, accelerate gastric emptying, promote colonic peristalsis, and reduce the incidence of dyspepsia and reflux [16, 17]. Indrio et al. [18] conducted a randomized, double-blind, controlled study of 30 preterm infants: 10 preterm infants were exclusively breastfed and the remaining 20 were fed formula; the formula-fed infants were randomly assigned to receive *L. reuteri* or a placebo for 30 days. The results showed that the number of reflux episodes in the *L. reuteri* group was significantly lower than that in the placebo group (*P* < 0.01). Moreover, the incidence of reflux episodes in the supplemented group resulted similar to that of the breastfed group. It demonstrated that oral supplementation with *L. reuteri* improved feeding tolerance in preterm infants. This is consistent with our findings. Our study demonstrated that *L. reuteri* was able to reduce severity and number of reflux episodes (GORD incidence was 8.88% in the intervention vs. 12.5% in the control group). We may speculate that this lead to a shorter TFF, thus improving the rapidity of the growth of formula-fed preterm infants. Our study also found that oral administration of *L. reuteri* may increase the daily defecation frequency of formula-fed preterm infants and shorten their hospital stays. Our study showed that no adverse effects occurred while formula-fed preterm infants were receiving *L. reuteri*, which was consistent with the results of Oncel et al. [19].

In the neonatal period, infections are common and are a major risk factor for neonatal death, especially septicemia, which has an insidious onset and rapid progression [20]. The digestive tract of preterm infants is hypoplasic, bacterial flora is less diverse, and colonization is delayed compared to full-term infants. Most digestive bacteria, including *Klebsiella*, *Enterobacter*, and *Clostridium*, are potentially pathogenic and can trigger digestive tract injury. Digestive tract injury combined with the deficiency of the innate immune system increases the probability that pathogens will spread throughout the entire body and cause a systemic infection [21]. Probiotics influence the functions of various immune cells, such as lymphocytes and dendritic cells, by direct or indirect regulation and they play a role in immune regulation and control of inflammation progression. *L. reuteri* DSM 17938 can ferment in vivo to produce acetic acid and reuterin. Acetic acid can lower the pH in vivo and it has a strong antibacterial effect on many pathogens; reuterin can cause oxidative stress in pathogens and effectively resist bacteria [22, 23]. Valeur [5] reported that *L. reuteri* activated CD4+ Th-cells and coordinated other immune cells to regulate the immune response. Preidis et al. [24] showed that *L. reuteri* could significantly induce the production of immunoglobulin A, inhibit the adhesion of bacteria and viruses to epithelial cells, and neutralize toxins. A randomized controlled trial showed that *L. reuteri* significantly reduced the incidence of diarrhea and respiratory infections in preschoolers, as well as shortened the course of disease [25]. Oncel et al. [26] reported that *L. reuteri* could significantly reduce the incidence of septicemia in extremely low birthweight (ELBW) infants with a gestational age of less than 32 weeks (6.5% vs. 12.5%, *P* = 0.041). On the contrary, in our study, there were no statistical differences in the incidence of sepsis (4.44% vs. 8.33%, *P* > 0.05) or localized infection (6.67% vs. 8.33%, *P* > 0.05) between the two groups. These two different results may be related to different sub-population, gestational age, birth weight or feeding patterns. Whether *L. reuteri* can prevent infection should be verified with larger sample sizes in future research.

The main pathogenesis of NEC include immature intestinal development, imperfect intestinal flora, formula feeding, and circulation disorders. The establishment of the microecological environment in the gastrointestinal tract is significant for maintaining the stability of the body environment and inhibiting intestinal inflammation. Breastfeeding is currently the only recognized protective factor against NEC. Research has confirmed that probiotics could effectively prevent and reduce the incidence of NEC in preterm infants [27, 28]. *L. reuteri* can improve the intestinal microecological environment, maintain the integrity of the gastrointestinal mucosal barrier, directly or indirectly regulate a variety of immune cell functions, and control the progression of intestinal inflammation [29]. Liu et al. [30, 31] found that *L. reuteri* could significantly down-regulate the level of tumor necrosis factor alpha by regulating TLR2, TLR4, and NF-кB signaling pathways in the intestine and reduce the incidence and severity of experimental NEC in rats. Hunter et al. [32] retrospectively analyzed 311 ELBW infants (79 cases with oral *L. reuteri* administration) and found that the incidence of NEC was significantly reduced in neonates receiving *L. reuteri* (15.1% vs. 2.5%, *P* = 0.0475). Our study showed that the incidence of NEC in the intervention group was 2.22% (1 case, stage IIA), while the incidence of NEC in the control group was 10.42% (5 cases in total: 3 cases in stage IIB, 2 cases in stage IIIA), which showed no statistically significant difference between the 2 groups (*P* > 0.05). More research is needed to confirm if oral administration of *L. reuteri* can reduce the incidence and severity of NEC in formula-fed preterm infants.

## Conclusion

In summary, *L. reuteri* DSM17938, as a safe microecological preparation, can reduce daily reflux, shorten the TFF; improve early feeding tolerance; accelerate increases in body weight, body length, and head circumference; promote growth; increase the frequency of defecation; and shorten the length of hospital stay in formula-fed preterm infants. Large sample sizes and multicenter studies are needed to confirm if oral administration of *L. reuteri* can reduce the incidences of nosocomial infection and NEC.

## Data Availability

The datasets generated and analysed during the current study are available in the Harvard Dataverse repository, 10.7910/DVN/XAEK0F

## Abbreviations

HC: Head circumference
*L. reuteri*: *Lactobacillus reuteri*
NEC: Necrotizing enterocolitis
TFF: Time to full enteral feedings
